# A Public Health Approach to Hepatitis C Control in Low- and Middle-Income Countries

**DOI:** 10.1371/journal.pmed.1001795

**Published:** 2015-03-10

**Authors:** Amitabh B. Suthar, Anthony D. Harries

**Affiliations:** 1 South African Centre for Epidemiological Modelling and Analysis, University of Stellenbosch, Stellenbosch, South Africa; 2 International Union Against Tuberculosis and Lung Disease, Paris, France; 3 Department of Infectious and Tropical Diseases, London School of Hygiene & Tropical Medicine, London, United Kingdom

## Abstract

In light of new treatment regimens for hepatitis C, Amitabh Suthar and Anthony Harries outline a wider public health approach for tackling the disease.

**Summary Points**

- New oral short-duration regimens using direct-acting antiviral medicines for hepatitis C virus (HCV) have the potential to facilitate treatment and improve outcomes.
- Translating scientific advances into reduced disease burden requires well-designed programmes encompassing prevention, screening, treatment, and strategic information.
- Engagement from countries, civil society, donors, and policymakers is needed to generate political commitment, mobilise resources, and reduce diagnostic and medicine costs for HCV.
- Countries should estimate the resources required to implement planned HCV prevention, screening, and treatment strategies and their expected health, societal, and financial benefits to mobilise domestic and international funding.
- Countries could integrate HCV prevention, screening, treatment, and strategic information into HIV/AIDS programmes for financial, infrastructural, and health workforce efficiencies.

## Introduction

An estimated 130–150 million people live with hepatitis C virus (HCV) [1]. A significant number of people with HCV will progress to chronic disease, hepatocellular carcinoma, and death [2]. Approximately 80 million people live with chronic HCV infection, and in 2013 an estimated 700,000 people died from HCV [3,4]. More than 80% of the HCV burden is in low- and middle-income countries [5]. Since there is no vaccine for HCV, prevention strategies currently rely on limiting exposure to the virus. The six different HCV genotypes vary geographically and affect susceptibility to some treatment regimens [6]. Recently, there have been significant scientific advances for treatment of HCV that have led to substantial interest in delivering HCV services through national programmes [7].

The first generation of direct-acting antivirals (DAAs) can cure specific genotypes when combined with interferon and ribavirin for 6–12 months [8–10]. Unfortunately, genotyping is unavailable at lower tiers of most health systems, interferon and ribavirin are difficult to tolerate, and interferon is an injectable medicine that requires administration by health care staff and a cold supply chain for national distribution. Treatment with some second-generation DAAs is associated with high rates of sustained virological response across genotypes and does not require interferon and ribavirin [8–10]. Patient adherence is likely to be high with second-generation DAAs because they are taken orally, are well-tolerated, and can be administered through fixed-dose combinations. Moreover, they can potentially be decentralised to peripheral health facilities with non-specialised physicians and nurses because of simple prescribing patterns, a short 3-month treatment course, and limited laboratory requirements.

There is an opportunity to translate scientific advances into effective public health programming; however, significant challenges remain. For example, unlicensed health technologies may perform poorly and thus waste scarce financial resources. Inappropriate use of drugs could lead to population-level resistance and reduce treatment options. Failing to implement a robust prevention strategy may lead to high levels of ongoing transmission. Finally, relying on patients to pay for HCV treatment out-of-pocket could induce inequities in which poor and marginalised populations cannot access essential HCV medicines.

## Programme Development

The goal of viral hepatitis programming should be to decrease hepatitis-related morbidity, mortality, and transmission. Such efforts require global, regional, and national stakeholders to develop high-level political commitment, mobilise resources, and propose targets [11]. Since many countries lack the necessary funding to provide HCV-related services, global stakeholders could further ensure that new treatment regimens are affordable for low- and middle-income countries. For example, some stakeholders aim to reduce HCV medicine costs through market shaping, advocacy, and patent opposition [12–16]. The Global Fund for AIDS, Tuberculosis and Malaria (GFATM), the President’s Emergency Plan for AIDS Relief (PEPFAR), and the President’s Malaria Initiative have been paramount in reducing HIV-, tuberculosis-, and malaria-related mortality globally. While limited provisions by the GFATM to finance HCV/HIV-co-infection screening and treatment in selected countries are encouraging, thus far there has not been a global financing initiative to reduce the burden of HCV in low- and middle-income countries [17].

**Fig 1:**
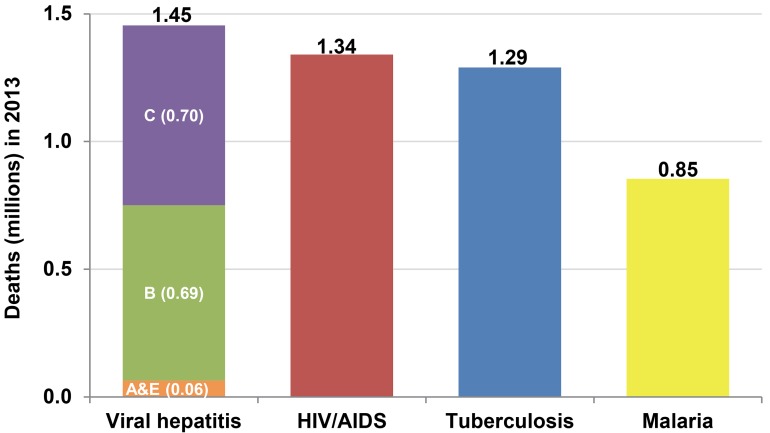
Number of deaths due to major communicable diseases in 2013 [4]. Viral hepatitis deaths include those related to acute viral hepatitis, liver cancer secondary to hepatitis B and hepatitis C, and cirrhosis of the liver secondary to hepatitis B and hepatitis C.

An important first step to mobilise support for a global financing initiative is the development of an investment case [18]. This would communicate to donors that viral hepatitis is a global epidemic that requires the same attention and resources as other major communicable diseases (Fig. 1). To facilitate discussions with national development partners, countries should estimate the financial, human, and physical resources required to implement planned HCV prevention, screening, and treatment strategies and their expected benefits on population health, society, and the economy. This information will help ministries of health explore external and domestic sources of funding, such as national and local budgets, national health insurance, and household out-of-pocket payments. Given the need to fund multiple public health needs, countries should conduct studies to determine the optimal resource allocation across diseases to improve the health and overall development of their populations.

There are ongoing efforts to increase the absolute number of skilled and motivated health workers [19]. While these long-term strategies begin to improve the human resource crisis, countries with current absolute health care worker shortages may need to consider task sharing—with training and supervision—to community- and peripheral-based health workers in order to expand access to HCV screening and treatment, respectively. It is important for countries to also examine opportunities for programmatic synergies. Specifically, rather than creating a separate programme for viral hepatitis, an integrated programme for HIV/AIDS and viral hepatitis could provide financial, infrastructural, and health workforce efficiencies. For example, the HIV laboratory infrastructure could diagnose and monitor HCV; preventing HIV through safe blood, harm reduction, and infection control also prevents HCV; HIV surveillance systems could generate essential HCV data; increasing domestic antiretroviral production capacity could improve access to HCV medicines and the HIV/AIDS workforce and health facilities can deliver essential HCV services. This approach would lead to integrated health programming and services [20–22].

## Prevention

HCV is transmitted through bodily fluids (e.g., blood, genital secretions, and cerebrospinal, amniotic, peritoneal, or pleural fluids). HIV co-infection may increase HCV viraemia and therefore increase the risk of transmission [23,24]. In contrast to hepatitis B, there is no vaccine for HCV, and its identification is a research priority. Effective prevention of HCV may therefore depend on reducing exposure to the virus through successful implementation of prevention interventions tailored to local transmission dynamics and expanded access to curative treatment, which reduces the amount of circulating virus in the community.

In many low- and middle-income countries, it appears that health care–related exposures, such as blood transfusion and percutaneous needlesticks, as well as injecting drug use are the most important modes of HCV transmission [25–29]. To prevent transmission through blood transfusion, blood and blood products must be universally screened [30]. Countries must screen for HCV antibodies, should screen for HCV antibody and antigen when combination antibody-antigen assays are available, and can screen for HCV RNA depending on available finances, laboratory infrastructure, and HCV incidence in donors [30]. Preventing percutaneous transmission in health care settings requires reducing unnecessary injections and implementing infection control measures, including exclusive use of therapeutic syringes with features to prevent reuse and injuries as well as safe handling and disposal of sharps and waste [31]. Legislation mandating these measures may improve implementation in both public and private health care facilities [32]. Expansion of harm reduction, including needle and syringe programmes and opiate substitution therapy, is needed to reduce HCV risk among people who inject drugs (PWID) [33].

Mother-to-child HCV transmission is approximately 6% in women without HIV and 11% in women with HIV [23]. There are currently no interventions to reduce HCV risk through this mode of transmission. There is an urgent need to evaluate the safety and effectiveness of DAAs during pregnancy and labour as they could reduce the concentration of virus in the mother and reduce transmission risk [34]. Although there is limited evidence of heterosexual HCV transmission [35], emerging data suggest potential for HCV transmission in men who have sex with men (particularly in the context of HIV co-infection) [24]. Condoms can be used to decrease sexual transmission risk. Finally, health education and training can help reduce transmission during nonmedical (e.g., commercial barbering, body piercing, tattoos, and traditional circumcision) and occupational (e.g., health care and sanitation worker) exposures.

Increasing coverage of treatment has reduced the incidence of tuberculosis and HIV in some settings [36,37]. Based on the simple principle of people being noninfectiousness after they are cured, there is potential for rapid expansion of DAAs to decrease population transmission of HCV [38]. Importantly, studies in generalised (i.e., national prevalence ≥1%) and concentrated (i.e., national prevalence <1% and prevalence in specific populations ≥5%) HCV epidemics indicate that DAA expansion in combination with other prevention interventions, which include universal access to safe blood, infection control, and harm reduction, yields the largest reduction in disease burden [39,40].

## Screening

Diagnosing hepatitis C requires serologic testing for HCV antibodies. An accurate, thermostable, and affordable rapid test is needed to improve access to HCV screening in health and community systems for all people with HCV, including those co-infected with HIV [41]. Since some of those initially infected will spontaneously clear the infection, nucleic acid amplification of RNA is needed to confirm chronic HCV infection. In settings where laboratories are used for HCV antibody screening, reflex HCV RNA testing after antibody is detected can ensure prompt HCV RNA results for patients and providers [42]. HCV core antigen may be an alternative to HCV RNA for screening in some settings [43]. Valid algorithms need to be developed to guide use of HCV biomarkers to diagnose HCV in different epidemiological contexts.

Many low- and middle-income countries have low diagnosis rates, and the majority of those infected remain undiagnosed until they develop serious liver disease [44]. Expanded HCV testing could achieve early diagnosis, but this has to be linked to care in order to reduce HCV-related morbidity, mortality, and transmission. Given the limited resources available for population screening efforts, epidemiological data should guide who should be prioritised for testing. For example, in the United States, where there is a concentrated HCV epidemic, substantial effort is needed to screen people who may have been exposed to unsafe blood (i.e., people born from 1945–1965, whose HCV prevalence is approximately 5-fold larger than other birth cohorts, and people who have haemophilia) and key populations (i.e., people at high HCV risk due to specific behaviours, such as PWID, sex workers, men who have sex with men, and people in prisons and other closed settings of detention) [45,46]. HCV testing services could reach the 1945–1965 birth cohort through systematic screening using birth dates in health care facilities, while risk-based screening in health care facilities and mobile testing could reach key populations. Key populations may also benefit from simultaneous screening of other diseases which share common transmission routes, e.g. HIV and hepatitis B. In Egypt, where there is a generalised HCV epidemic and HCV prevalence is highest in people with ongoing or a past history of medical injections, screening is provided in hospitals, all health facilities, workplaces, and using mobile testing [47,48]. Given the dramatic need to increase knowledge of HCV status in most settings, community health workers may need to be trained to provide counselling and rapid serologic tests, while nonspecialised physicians and nurses may need to be trained on interpreting HCV RNA or core antigen results for diagnosing chronic HCV infection.

Encouraging people to discover their HCV status and reduce transmission can be done through targeted use of media, educational campaigns, World Hepatitis Day, and other approaches. WHO’s 5C’s human rights approach for HIV screening may provide important guiding principles for HCV screening (requiring informed Consent, Confidential in nature, pre- and post-test Counselling, Correct test results, and Connections to care) [49]. Counselling may focus on how to reduce the risk of acquiring or transmitting HCV, the need for repeat testing (for key populations at high risk of acquiring HCV), and the possible benefits of therapy, among other key messages.

## Treatment

All people with confirmed chronic HCV infection should be offered high-quality care as soon as possible. In addition to essential elements of primary care, this should also include screening and management of alcohol use to prevent progression to cirrhosis [50]. Countries will also need to decide when to start HCV treatment. Modelling suggests that treatment early in the course of HCV disease for all or specific populations could prevent disease progression and onward transmission [39,40,51]. However, not all people with chronic HCV infection will progress to fibrosis [2]. Moreover, transmission has been documented among people recently cured who lacked access to prevention services [52]. Research is needed to determine cost-effective eligibility criteria for both key and other populations that maximise reductions in HCV-related morbidity, mortality, and transmission in different epidemiological contexts.

Recent WHO guidelines recommend prioritising treatment among people with advanced fibrosis or cirrhosis in order to prevent liver cancer and death [53]. HCV has been shown to increase mortality in people with HIV, while HIV has been shown to accelerate HCV disease progression [54,55]. Therefore, people with HIV/HCV coinfection may also warrant treatment prioritisation. The METAVIR system can be used to estimate the stage of HCV disease through liver biopsy and identify individuals needing treatment [56]; however, liver biopsy is expensive, subject to sampling errors, invasive for patients, and can lead to complications such as infections, excessive bleeding, pain, or accidental injury to other organs. Alternative non-invasive staging criteria that use liver function tests and platelet counts to determine the degree of liver fibrosis, such as the aminotransferase-to-platelet ratio index (APRI) and the Fibrosis-4 score (FIB-4), may be a more useful approach for gauging treatment eligibility across different tiers of health systems [57]. Systems should be put in place to retain and monitor people who do not initiate treatment immediately.

Countries will also need to have a careful discussion on what drugs should be used to treat HCV. Global and national guidelines can help ensure the rational selection of medicines in national essential medicine lists and health insurance. The regimen should, at a minimum, be highly effective, well-tolerated, short-duration, and affordable (Table 1). Pangenotypic regimens, i.e., regimens with similar efficacy across genotypes, eliminate the need for relatively expensive and inaccessible genotyping. For training, financial, and supply chain reasons, a single regimen should be used for all populations (including paediatrics, pregnant women, and key populations such as PWID). Evaluating the safety and effectiveness in these populations is a research priority, since these populations have been systematically excluded from most clinical trials of new DAAs. Current WHO guidelines recommend sofosbuvir and ribavirin, with or without pegylated interferon, as the preferred regimen for HCV treatment [53]. Major toxicities with ribavirin, such as anaemia and foetal abnormalities, may indicate a need to consider alternative regimens as they become available. Routine public health programming activities should measure the incidence of these and other life-threatening adverse events [58].

**Table 1:** Properties of second-generation oral anti-HCV agent classes [8,10,59].

	NS5B nucleos(t)ide inhibitor	NS5A inhibitor	NS3/4A protease inhibitor	NS5B non-nucleoside inhibitor
Pangenotypic	+++	++	++	+
Potency	+++	++	++	+
Potential for price reductions	+++	+++	+++	-
Major drug interactions	++ Rifampicin could decrease concentrations through P-glycoprotein induction	-	+++ Rifampicin, efavirenz, and HIV protease inhibitors could decrease concentrations through P450 induction	-
Studied in pregnant women, paediatrics, and PWID	+	+	+	+
Treatment limiting toxicities	+	+	++ Gastrointestinal distolerance and anaemia	+
Barrier to resistance	+++	++	++	+
Agents (bold indicates licensed, italicised indicates phase III, and normal formatting indicates phase II)	**Sofosbuvir** (Gilead), *Mericitabine* (Roche), VX-135 (Vertex)	**Ledipasvir** (Gilead), **Ombitasvir** (AbbVie), *Daclatasvir* (Bristol-Myers Squibb), *Elbasvir* (Merck), *GS-5816* (Gilead), *ABT-530* (AbbVie), PPI-668 (Presido)	**Simeprevir** (Janssen), **Paritaprevir/ritonavir** (AbbVie), *Asunaprevir* (Bristol-Myers Squibb), *Grazoprevir* (Merck), *Danoprevir* (Roche), *Faldaprevir* (Boehringer-Ingelheim), *ABT-493* (AbbVie), *Vaniprevir* (Merck), GS-9451 (Gilead)	**Dasabuvir** (AbbVie), *Beclabuvir* (Bristol-Myers Squibb), *GS-9669* (Gilead)

+ indicates low level, ++ indicates moderate level, +++ indicates high level, and - indicates unknown level. US Food and Drug Administration license dates current as of 31 December 2014.

Abbreviations: NS, nonstructural protein.

Legal and financial barriers impose a significant obstacle in licensing and providing DAAs in low- and middle-income countries. There are various policy options to expand access to HCV curative treatment by reducing cost [60]: (1) voluntary licensing (patent holders voluntarily licensing generic manufacturing and fixed-dose combinations in defined territories), (2) differential pricing (patent holders developing different prices based on the countries’ willingness and ability to pay), (3) pre- and post-grant opposition (third parties disputing the validity of filed patents in countries that have implemented flexibilities of the World Trade Organisation Agreement on Trade-Related Aspects of Intellectual Property Rights [TRIPS]), or (4) compulsory licenses (countries either importing or locally producing the medications by implementing TRIPS flexibilities). Thus far, manufacturers of sofosbuvir and daclatasvir have proposed the use of voluntary licensing for some low- and middle-income countries and differential pricing for others [61,62]. While this approach proposes a price reduction for 12 weeks of sofosbuvir from approximately US$84,000 to US$900 for certain countries [62], the production price remains substantially less [59]. Since production prices for other DAAs are similar to sofosbuvir, there is an opportunity to achieve further price reductions in providing HCV treatment [59]. Pre-grant opposition led India’s patent office to reject sofosbuvir’s base compound patent because of patent innovation requirements [63]. Generic production, generic competition, and price reductions could ensue if the prodrug patent is also rejected [16]. To facilitate negotiation with generic manufacturers and drive economies of scale, global financing mechanisms and governments will need to procure large quantities of the drugs. In the absence of price reductions, DAAs may not be a cost-effective treatment option over continued use of pegylated interferon and ribavirin, particularly for easier to treat genotypes.

Health systems aim to deliver accessible, acceptable, and affordable services. To improve accessibility and linkage to care, HCV treatment should be integrated into health services that will provide HCV screening, such as hospitals, primary health facilities, drug-dependence treatment centres, and HIV/AIDS facilities. Given the absolute shortage of health care providers, training and supervision from specialised physicians to non-specialised physicians and nurses to stage patients clinically, initiate treatment, and maintain treatment until cure may be required. The short duration of HCV treatment with newer DAAs could make directly observed treatment worth considering in some settings. Countries will also need to implement central procurement and supply chain management systems encompassing antiviral registration, forecasting, procurement, storage, and distribution to prevent stockouts and reduce cost [64].

Sustained virological response (SVR), i.e., the absence of HCV RNA 6 months after treatment completion, could be the most critical marker of treatment success [65]. Many low- and middle-income countries have limited laboratory infrastructure and lack access to HCV RNA for confirming chronic HCV infection and determining treatment outcome. National laboratory strategic plans are guiding laboratory development as part of health systems strengthening [66]. Using dried blood spots may help increase access to HCV RNA technology [67]. This approach could be linked to established systems, such as early infant diagnosis of HIV and antiretroviral therapy monitoring [68]. High-quality, low-cost, point-of-care RNA tests may also be available in the future [12,15]. HCV core antigen may be a qualitative alternative to quantitative RNA for measuring treatment response [69]. Health technology assessments by national panels can ensure rational selection of HCV platforms based on available resources and health system organisation.

## Strategic Information

Countries will have to collect and collate information relevant for developing and modifying HCV strategies. Integrating HCV into existing surveillance systems, such as population demographic and health surveys in generalised epidemics and integrated biological and behavioural surveillance for key populations in all epidemics, is needed to accurately measure HCV burden and transmission nationally, regionally, and in different subpopulations over time [70,71]. Although limited by biases and generalizability in many countries, routinely collected data can also provide useful information [72]. For example, blood banks, drug-dependence treatment centres, and antenatal facilities could provide prevalence estimates. Mortality could be estimated from civil and vital registration systems, verbal autopsy studies, cancer registries, cremation and burial services, and health facility data. Morbidity due to cirrhosis and hepatocellular carcinoma could be estimated from hospitals, health facilities, and hepatitis notification systems. Since acute HCV infection is largely asymptomatic, it is not routinely diagnosed in health facilities; incidence may need to be estimated by mathematical modelling or routinely screening representative cohorts for acute HCV infection through sentinel surveillance.

Expanding commonly used tools, such as OneHealth, to include HCV may help in projecting the impact and cost-effectiveness of different national strategies [73]. After developing national HCV strategies, countries will also need to measure progress in implementing them. If systematic reporting occurs, cases reported from sites providing HCV screening measured over national burden estimates can allow programmes to estimate case detection rates. Mandatory reporting has been used for other infectious diseases to ensure access to care and treatment services and might merit consideration for HCV [74]. Linkage to care could be measured by dividing the number of people who enrol into care by the number of people who are diagnosed with chronic HCV infection. Standardised patient monitoring systems should be used to monitor treatment response and life-threatening toxicities. Treatment outcomes could focus on five outcomes after completion of therapy: success, failure, lost-to-follow-up, death, and transferred in/out. Treatment success rates could be calculated from health registries using cohort analysis by dividing the number of people who achieve SVR over the number of people who initiate treatment. These indicators can be measured over a treatment cascade. Existing health indicators, such as access to safe blood, infection control practice, access to sterile syringes for PWID, and opiate substation therapy coverage, will help gauge success in prevention programming.

## Conclusions

Advances in therapeutic options for hepatitis C make improving its detection and treatment important (Box 1). Global stakeholders, including civil society, countries, donors, and policy makers, can ensure hepatitis C is a priority on global development agendas. Countries and partners should begin (1) collecting, analysing, and interpreting data to understand their epidemics and develop investment cases, (2) aggressively scaling up robust prevention strategies based on local transmission dynamics, and (3) providing systematic screening and treatment services initially prioritising those most in need. The major limiting steps are the high cost of treatment and the absence of affordable and reliable diagnostic tests; similar conundrums were faced 15 years ago by the HIV/AIDS community. The success of this community in driving major price reductions for lifelong antiretroviral therapy and achieving universal treatment access demonstrates the feasibility of reducing the cost of short-course HCV treatment and rapidly expanding its access as part of a comprehensive viral hepatitis response [75].

**Box 1. Key Actions for Creating a Public Health Approach to HCV**

**Programme development**

- Develop political commitment, mobilise resources, reduce costs of medicines and diagnostics, and support training programmes for peripheral and community-based health workers
- Estimate resources and benefits associated with an HCV response
- Develop a global financing mechanism for resource-constrained settings

**Prevention**

- Screen all donated blood for HCV
- Reduce unnecessary injections
- Implement infection control measures
- Provide clean needles and opiate substitution therapy to PWID

**Screening**

- Prioritise screening amongst the most affected populations, including those exposed to unsafe blood, key populations, and people living with HIV/AIDS
- Develop standardised diagnostic algorithms
- Use appropriate mix of health facility—and community-based approaches linked to HCV care and treatment

**Treatment**

- Use APRI or FIB-4 for gauging treatment eligibility
- Use effective, well-tolerated, short-duration, and affordable regimens in national guidelines
- Where possible, integrate treatment into HCV screening sites

**Strategic information**

- Integrate HCV into surveillance systems
- Measure access to safe blood, infection control practice, and harm reduction coverage
- Use national HCV estimates, case reporting, and treatment cohort analysis to measure HCV service outcomes across a cascade

Abbreviations: HCV, hepatitis C virus; PWID, persons who inject drugs; APRI, aminotransferase-to-platelet ratio index; FIB4, Fibrosis-4 score

## Abbreviations

APRI: aminotransferase-to-platelet ratio index
DAAs: direct-acting antivirals
FIB-4: Fibrosis-4 score
GFATM: The Global Fund for AIDS, Tuberculosis and Malaria
HCV: hepatitis C virus
NS: nonstructural protein
PEPFAR: the President’s Emergency Plan for AIDS Relief
PWID: people who inject drugs
SVR: sustained virological response
TRIPS: trade-related aspects of intellectual property rights
